# Attempts to Establish a Helicobacter bilis Biliary Tract Infection Model via Oral Administration of Helicobacter bilis

**DOI:** 10.7759/cureus.95008

**Published:** 2025-10-20

**Authors:** Taichiro Kosaka, Tomohiko Adachi, Hajime Imamura, Takashi Hamada, Hajime Matsushima, Takanobu Hara, Ayaka Kinoshita, Akihiko Soyama, Susumu Eguchi

**Affiliations:** 1 Department of Surgery, Nagasaki University Graduate School of Biomedical Sciences, Nagasaki, JPN

**Keywords:** biliary tract cancer, gallbladder, hamster model, helicobacter bilis, intrahepatic bile duct

## Abstract

Background

Although several studies have reported an association between *Helicobacter bilis* (*H. bilis*) and human biliary tract cancer, in vivo studies are necessary to further assess this phenomenon. In this study, we developed an animal model of *H. bilis *infection, determined whether oral exposure to* H. bilis *induces biliary carcinogenesis, and evaluated colonization and pathological changes.

Methods

Seven-week-old female Syrian golden hamsters were administered *H. bilis* via the intragastric route using a sonde and euthanized after two, four, 12, 24, and 48 weeks. We assessed the rate of bacterial colonization in the intrahepatic bile ducts and gallbladders, polymerase chain reaction (PCR) positivity rate using *H. bilis*-specific primers, carcinogenic or pathological alterations, and cholangitis scores.

Results

*H. bilis* was detected in the intestinal tract of hamsters but not in the biliary tract. PCR positivity was higher in the *H. bilis *group than in the control group at each time point, but not statistically significant. Histological alterations in the biliary system of the orally administered group were limited to gallbladder hyperplasia, with no evidence of carcinogenesis. The cholangitis scores were similar between the two groups.

Conclusions

In this study, the oral administration of *H. bilis* alone did not induce carcinogenesis. Because human epidemiological studies have revealed that *H. bilis* is involved in carcinogenesis, further research is needed to elucidate the relationship between *H. bilis* and human biliary tract cancer.

## Introduction

*Helicobacter* spp. have been reported to cause infections in humans and various animal species, including mice, rats, rabbits, dogs, and cats [1,2]. *Helicobacter bilis* (*H. bilis*), a gram-negative spiral bacillus, is ubiquitous in the gastrointestinal tract of humans and animals, where it can adapt and reproduce. *H. bilis* was formally described as a species in 1995 [3], following reports of related “flexispira”-like organisms in the late 1980s [4]. In mice, *H. bilis* colonizes the bile, liver, and intestines and is associated with multifocal chronic hepatitis and hepatocellular tumors [3].

The relationship between *Helicobacter pylori* (*H. pylori*), a member of the *Helicobacter* genus, and gastric cancer is well known, with numerous studies on the carcinogenesis mechanism of *H. pylori* and the benefits of its eradication in suppressing carcinogenesis [5-7]. However, *H. bilis* has also been reported to be associated with biliary tract diseases [8,9]. Specifically, we have reported its clinical association with intrahepatic stones and cholangiocarcinoma [10,11], as well as its association with abnormal pancreaticobiliary confluence [12]. Recently, Yamashita et al. [13] assessed the association between *H. bilis* and this type of clinical carcinogenesis in vitro. Persistent infection of the biliary epithelium provokes inflammatory responses, leading to cytokine production and oxidative stress-mediated DNA damage, thereby creating a milieu conducive to tumorigenesis [13].

Although these studies indicate an association between *H. bilis* and biliary tract carcinogenesis, all results were obtained from polymerase chain reaction (PCR)-based human in vitro studies. Additionally, research from Thailand highlights the need for more specific assays to advance the epidemiological understanding of *H. bilis* infection [14]. Therefore, we consider it essential to conduct long-term in vivo observations using an *H. bilis*-infected animal model to explore its association with biliary tract carcinogenesis. In addition, once this model is established, it may also contribute experimentally to cancer suppression through eradication, similar to the outcomes achieved with *H. pylori*. In this study, we aimed to develop a long-term observation model of *H. bilis* by administering live bacteria to healthy hamsters.

## Materials and methods

Culture preparation of *H. bilis*

*H. bilis* ATCC 51630 was cryopreserved on dry ice. Cells were incubated in 2.8% Brucella broth (Becton Dickinson, Franklin Lakes, NJ) with 1.5% agar (Wako Pure Chemical Industries, Osaka, Japan), 5% defatted sheep blood (Japan Biotest Laboratories, Tokyo, Japan), and 5% heat-inactivated fetal bovine serum (FBS) (Gibco, Gibco, Thermo Fisher Scientific, Waltham, MA) at 37°C in a microaerophilic atmosphere (3%-5% O2, 10% CO2). The microaerophilic atmosphere was established using an anaerobic jar containing a microaerophilic gas-generating pack (Mitsubishi Gas Chemical Company, Inc., Tokyo, Japan) and moistened paper. After seven days of culture, the cells were transferred to a liquid medium containing 2.8% Brucella broth and 10% heat-inactivated FBS for subsequent cell cultures. Subsequently, the cells were collected through centrifugation (1000 × g, 25°C, 10 minutes). The supernatant was discarded, and the bacterial pellet was washed with liquid medium and centrifuged again under similar conditions. The final pellet was resuspended in liquid medium, and the bacterial concentration was adjusted to an optical density at 600 nm (OD600) of 0.3. The suspension was dispensed into culture tubes and incubated at 37°C for 48 hours in a microaerophilic atmosphere.

Animal experiments

The animal experiments adhered to the guidelines of the Guide for the Care and Use of Laboratory Animals of the US National Institutes of Health. The protocol was approved by the Nagasaki University Animal Experimentation Ethics Committee (0704270579). A model of *H. bilis* infection was developed by oral intragastric administration of *H. bilis* to a group of seven-week-old female golden Syrian hamsters. Because the anatomy of the pancreas and biliary tract and the composition of bile in hamsters are similar to those in humans, we often use these animals for experimental studies [15,16]. In the intragastric administration group, *H. bilis* (1 × 108 colony-forming units/1 mL saline) was administered under general anesthesia using a gastric tube sonde. The control group was administered only 1 mL saline orally. Both groups received a single dose. For several model animals, sacrifice was performed on days 1 and 7 to observe and evaluate the actual engraftment status in the cecum and liver of *H. bilis*. The groups were further divided into six subgroups based on the observation period (two, four, 12, 24, and 48 weeks) and euthanized after each period. The detection rate of *H. bilis* in the gallbladder and liver tissues was assessed using Giemsa staining or PCR. Additionally, the relationship between histopathological findings - assessed using hematoxylin and eosin (H&E) staining - and the severity of cholangitis was assessed using our standard criteria, as follows: grade 0, no cholangitis; grade 1, mild infiltration of inflammatory cells around the bile duct; grade 2, severe infiltration of inflammatory cells around the bile duct; and grade 3, abscess formation in the liver [17].

PCR assessment

To avoid contamination, equipment treated with UV irradiation was used, and all procedures from DNA extraction to PCR were performed according to a unidirectional workflow. DNA was extracted from each tissue sample using a commercial DNA extraction kit (ISOTISSUE; Nippon Gene, Tokyo, Japan). Approximately 50 mg of each tissue sample was homogenized with a sterile metal pestle and processed according to the manufacturer’s instructions. Bile samples (approximately 1.5 mL) were pelleted by centrifugation at 10,000 × g for 10 minutes and processed in the same way as other tissue samples. One primer sets were used to detect Helicobacter DNA. Primer set C62/C12, specific for the 16S rRNA of *H. bilis* (C62: AGAACTGCATTTGAAACTACTTT; C12: GGTATTGCATCTTTGTATGT), amplifies a 638-bp fragment.

PCR was performed in a final volume of 25 μL containing 2 mmol/L MgCl₂, 200 μmol/L of each dNTP, 0.4 μmol/L of each primer, 1.25 U of Ex Taq DNA polymerase (TaKaRa Bio, Shiga, Japan), and 1 μL of template DNA, with the remaining volume adjusted with nuclease-free water. The amplification protocol consisted of an initial denaturation at 94°C for five minutes, followed by 40 cycles of denaturation at 94°C for one minute, annealing at 56°C for 30 seconds, and extension at 72°C for one minute, with a final extension at 72°C for seven minutes. PCR products (10 μL) were electrophoresed on 2.0% agarose gels, stained with SYBR Gold (Invitrogen, Carlsbad, CA), and visualized under UV light. All experiments were performed at least twice to confirm reproducibility. PCR products were purified using the QIAEX Gel Extraction Kit (Qiagen, Courtaboeuf, France) and sequenced using an ABI 377 DNA sequencer (Applied Biosystems, Foster City, CA). Sequence similarity searches were performed using the BLAST program (NCBI, Bethesda, MD) against the GenBank database (http://www.ncbi.nlm.nih.gov/genbank). DNA extracted from *H. bilis *ATCC 51630 was used as a positive control.

Endpoints and statistical analysis

The primary endpoint of this study was the incidence rate of the biliary tract cancer. Secondary endpoints were PCR positivity of the *H. bilis* or cholangitis score in the specimen. Standard univariable statistical tests, the two-tailed Fisher’s exact test was used to compare categorical variables between the groups. Statistical significance was set at p < 0.05. All analyses were performed using BellCurve (Social Survey Research Information Co., Ltd., Tokyo, Japan) for Excel (Microsoft Corporation, Redmond, WA).

## Results

Detection of *H. bilis* in hamster specimens

**Figure 1:**
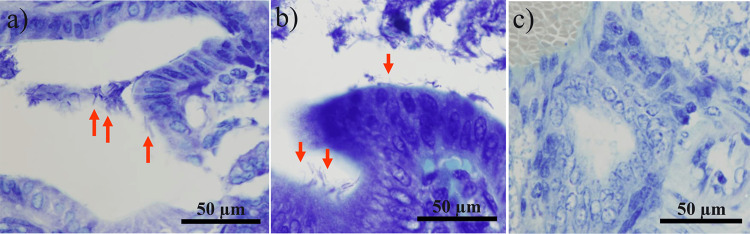
Giemsa staining of the cecum on days 1 (a) and 7 (b) in the experimental model. Arrowheads indicate bacteria. Giemsa staining of the liver on day 7 (c). Specimens were observed under a microscope (×400).

Giemsa stain-positive bacilli were observed in the cecum of the experimental hamster model on days 1 and 7 (Figure 1a and Figure 1b, respectively). In contrast, *H. bilis* was not detected in the liver on either day (Figure 1c).

PCR assessment

**Figure 2:**
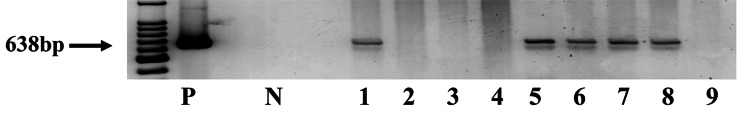
Polymerase chain reaction-based detection of positive rates in the 24-week experimental model. N: Negative control; P: positive control. The numbers represent each sample number. Five out of nine cases were positive.

Figure 2 illustrates the PCR findings for liver samples from *H. bilis *model hamsters at 24 weeks, with five out of nine samples testing positive at 638 bp. The PCR results are summarized in Table 1. Although the *H. bilis* group exhibited a higher PCR positivity rate compared to the control group, no significant differences were observed at any of the time points.

**Table 1:** Detection rate of Helicobacter bilis in the biliary tract using polymerase chain reaction (PCR).

	Weeks	Control	*Helicobacter bilis* model	p
PCR positive	2	4/10 (40%)	6/10 (60%)	0.65
	4	2/10 (20%)	2/10 (20%)	1.00
	12	2/9 (22%)	6/8 (75%)	0.09
	24	1/8 (13%)	5/9 (56%)	0.12
	48	2/8 (25%)	3/6 (50%)	0.33
	Total	11/45 (24%)	27/43 (63%)	-

Pathological examination

**Figure 3:**
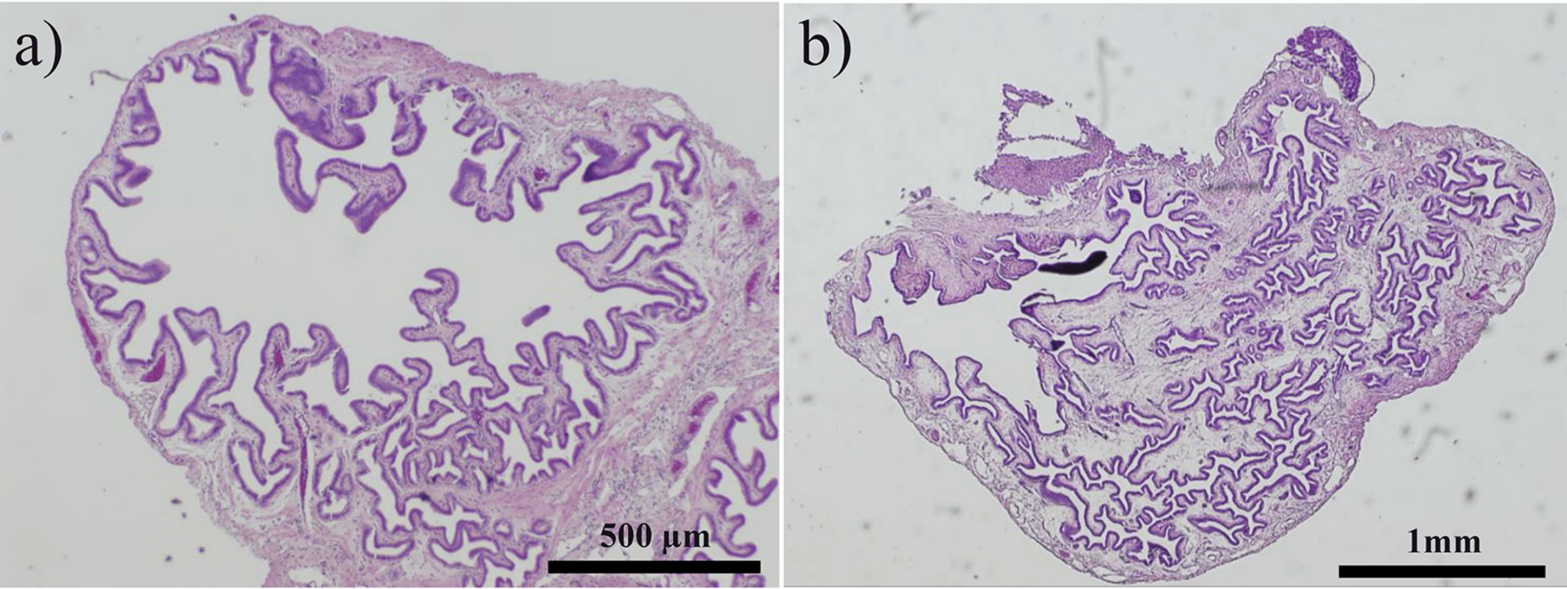
Gallbladder mucosal hyperplasia observed in 33% (3/9 cases) of the Helicobacter bilis group at 24 weeks (a, b), with no evident malignant lesions.

The gallbladders were collected from each hamster upon euthanasia and subjected to H&E staining. No obvious cancerous lesions, including in situ lesions, were observed in the gallbladder during any observation period, even in the *H. bilis* model. Hyperplasia was observed in only a few cases (Figures 3a-3b), with its incidence summarized in Table 2. In the model and control groups, hyperplasia increased with age, albeit not significantly. Additionally, no histological neoplasia, including hyperplasia, was observed in the intrahepatic bile ducts of both groups.

**Table 2:** Incidence of hyperplasia in the gallbladder.

	Weeks	Control	*Helicobacter bilis* model	p
Hyperplasia in the gallbladder	2	0/10 (0%)	0/10 (0%)	1.00
	4	0/10 (0%)	0/10 (0%)	1.00
	12	0/9 (0%)	0/8 (0%)	1.00
	24	1/8 (13%)	3/9 (33%)	0.66
	48	1/8 (13%)	1/6 (17%)	1.00
	Total	2/45 (4%)	4/43 (9%)	-

**Figure 4:**
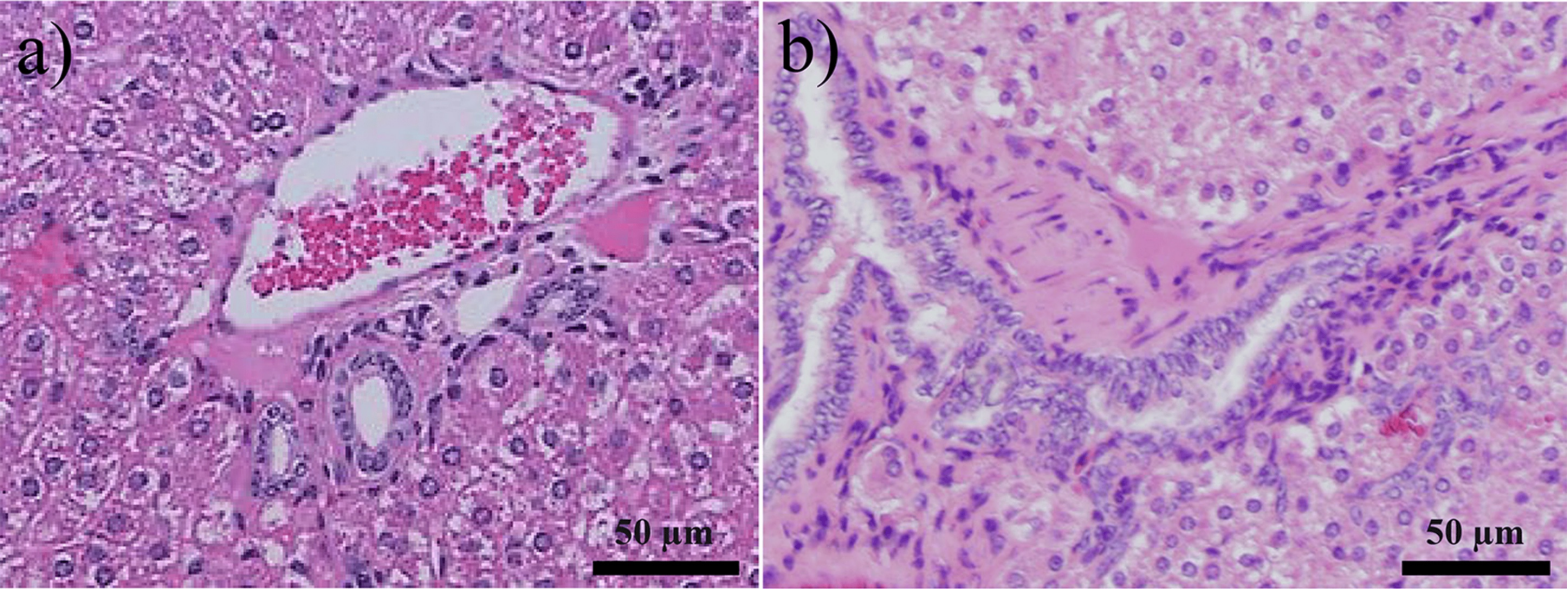
Hematoxylin and eosin staining of the liver. No inflammatory cell infiltration around the bile duct in the control group (score: 0) (a). Mild inflammatory cell infiltration around the bile duct (score: 1) (b).

**Figure 5:**
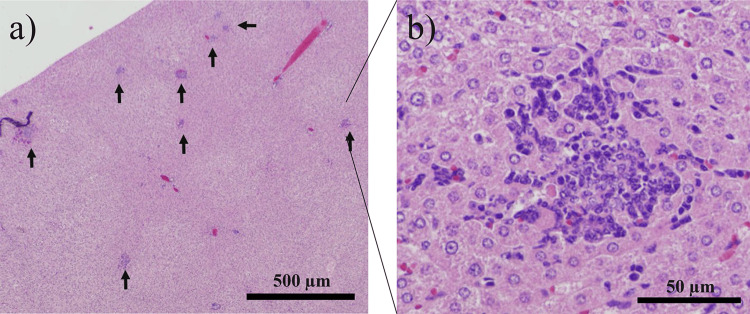
Microabscesses observed in hematoxylin and eosin-stained liver tissues. Black arrow: microabscess (×20) (a), magnified view of the abscess (×400) (b).

To assess whether *H. bilis* contributes to inflammation-induced carcinogenesis, cholangitis scores around the intrahepatic bile ducts were compared between the two groups. Figure 4a illustrates a cholangitis score of 0, whereas Figure 4b illustrates a score of 1. The scores were assigned based on the severity of cholangitis, which is described in the Materials and Methods. Multiple micro-liver abscesses were also observed in the *H. bilis* model (Figures 5a-5b). Table 3 summarizes the study results. No significant differences in cholangitis scores were observed between the two groups.

**Table 3:** Cholangitis score of the 24-week model in each group.

Score	Control (n = 8)	*Helicobacter bilis* model (n = 9)	p
0	2 (25%)	1 (11%)	0.56
1	6 (75%)	8 (88%)	
Average score	0.75	0.89	-

## Discussion

In cancer research, in vivo models have emerged as significant tools for studying and addressing crucial questions related to cancer [18]. Models that are used to study cancer within a living organism offer valuable insights into tumor behavior, progression, and potential therapeutic approaches [19] because they can mimic the complex interactions and dynamics of tumors within a living system. From this perspective, we believe that an infection model is necessary to determine the relationship between biliary tract cancer and *H. bilis* infection. Previous studies on the *H. bilis* infection model have provided valuable insights into its relationship with inflammatory bowel disease [20]. Maggio-Price et al. [21] demonstrated that *H. bilis* and *Helicobacter hepaticus* infection accelerates and delays the development of colitis in multiple drug-resistant-deficient (mdr1a-/-) mice with oral administration similar to ours. Atherly et al. [22] reported that *H. bilis* infection alters the mucosal bacteria population and modulates colitis development in mice [22]. They also demonstrated that colitis development in colonized mice was associated with temporal alterations in the composition and spatial distribution of mucosal microbiota. In contrast, experiments that did not use the developed model but assessed *H. bilis*-induced biliary infection and liver pathology in old hamsters were also reported [23]. In this experiment, the progression of liver lesions, including chronic hepatitis, hepatocellular dysplasia, fibrosis, and bile duct hyperplasia, was observed in naturally *H. bilis*-infected hamsters. To the best of our knowledge, our study is the first to develop a long-term observation model of infection with *H. bilis*. We previously assessed biliary tract carcinogenesis using N‐nitrosobis (2‐oxopropyl) amine (BOP) following choledochojejunostomy [15,16,24,25]; in this study, although PCR analysis revealed the presence of bacteria, no biliary tract carcinogenesis was observed. BOP was not used in this study because we aimed to confirm whether spontaneous carcinogenesis can occur, which is analogous to the findings of epidemiological studies. This will be explored in future studies.

Because *H. bilis* is endemic to the intestinal tract of rodents, a small amount was detected by PCR even in the control group. In this study, *H. bilis* DNA was detected in the liver tissue of the *H. bilis* group using PCR. However, microscopic examination of the biliary tract did not reveal *H. bilis* infection. This finding aligns with those of previous human epidemiological studies [10-12]. *H. bilis* infection in the bile or bile duct tissue has only been identified using PCR with DNA-specific primers. Additionally, the isolation of *H. bilis* from human biliary tissue using tissue culture or microscopy did not identify infection foci. These findings led us to hypothesize that biliary tract carcinogenesis can be caused by *H. bilis* DNA alone in the absence of *H. bilis* organisms in the intrahepatic bile ducts. For example, DNA from dead bacteria in the intestinal tract may reach the liver through enterohepatic circulation. Normally, the immune response suppresses cancer progression. Toll-like receptors (TLRs) play a crucial role in innate immunity against bacteria. Specifically, TLR9 recognizes unmethylated CpG sequences in the DNA of pathogenic microorganisms that have been internalized into the cell and initiates immune signaling through the myeloid differentiation primary response 88/nuclear factor kappa B pathway, thereby activating the immune response cascade [26].

The association between biliary tract cancer and chronic inflammation is well established. Therefore, for biliary tract carcinogenesis, the following hypotheses are proposed: 1) DNA released from dead *H. bilis* in the intestinal tract enters the liver through enterohepatic circulation via the portal vein; 2) a continuous TLR9-mediated immune response occurs at the same site of the immune response; and 3) chronic inflammation results in biliary tract carcinogenesis. We are currently conducting research to test these hypotheses.

This study has several limitations. First, although we used Syrian golden hamsters because of their anatomical and physiological similarities to the human biliary system, extrapolation of the findings to human biliary carcinogenesis remains limited. Second, oral administration of *H. bilis *alone may not fully reproduce the multifactorial process of human biliary tract cancer, in which co-factors such as chemical carcinogens, chronic inflammation, or genetic predisposition are often involved. Third, the sample size in each subgroup was relatively small, which may have limited the statistical power to detect subtle pathological changes. Fourth, although PCR allowed sensitive detection of *H. bilis* DNA, the absence of bacterial localization by microscopy or culture limits the interpretation of whether active colonization occurred in the biliary tract. Finally, the observation period, although extended up to 48 weeks, may still have been insufficient to observe carcinogenesis, which could require longer exposure or additional carcinogenic stimuli. These limitations should be addressed in future studies by combining *H. bilis* infection with established chemical or surgical carcinogenesis models, increasing the sample size, and extending the duration of observation.

## Conclusions

In conclusion, *H. bilis *DNA was detected in the liver using PCR; however, carcinogenesis was not observed even after long-term observation. Although this study presents negative results, we believe that they will aid future cancer research by demonstrating that this method significantly increases *H. bilis* DNA detection but does not result in carcinogenesis. Because human epidemiological studies have revealed that *H. bilis* is involved in carcinogenesis, further research is needed to elucidate the relationship between *H. bilis* and human biliary tract cancer.
